# Point-of-care diagnosis of periodontitis using saliva: technically feasible but still a challenge

**DOI:** 10.3389/fcimb.2015.00065

**Published:** 2015-09-03

**Authors:** Suk Ji, Youngnim Choi

**Affiliations:** ^1^Department of Periodontology, Anam Hospital, Korea UniversitySeoul, South Korea; ^2^Department of Oral Microbiology and Immunology, School of Dentistry and Dental Research Institute, Seoul National UniversitySeoul, South Korea

**Keywords:** periodontitis, point-of care testing, saliva, bacteria-derived biomarkers, host-derived biomarkers

## Abstract

Periodontitis is a chronic inflammation of the periodontium caused by persistent bacterial infection that leads to the breakdown of connective tissue and bone. Because the ability to reconstruct the periodontium is limited after alveolar bone loss, early diagnosis and intervention should be the primary goals of periodontal treatment. However, periodontitis often progresses without noticeable symptoms, and many patients do not seek professional dental care until the periodontal destruction progresses to the point of no return. Furthermore, the current diagnosis of periodontitis depends on time-consuming clinical measurements. Therefore, there is an unmet need for near-patient testing to diagnose periodontitis. Saliva is an optimal biological fluid to serve as a near-patient diagnostic tool for periodontitis. Recent developments in point-of-care (POC) testing indicate that a diagnostic test for periodontitis using saliva is now technically feasible. A number of promising salivary biomarkers associated with periodontitis have been reported. A panel of optimal biomarkers must be carefully selected based on the pathogenesis of periodontitis. The biggest hurdle for the POC diagnosis of periodontitis using saliva may be the process of validation in a large, diverse patient population. Therefore, we propose the organization of an International Consortium for Biomarkers of Periodontitis, which will gather efforts to identify, select, and validate salivary biomarkers for the diagnosis of periodontitis.

## Usefulness of salivary diagnostics for periodontitis

Periodontitis is a chronic inflammation of the periodontium caused by persistent bacterial infection that leads to the breakdown of connective tissue and bone (Ji et al., 2014). Due to its chronic nature, periodontitis progresses without causing severe discomfort in the oral cavity, and patients often seek professional care only after the periodontium is considerably destroyed. Thus, there is a need to diagnose periodontitis in its initial stages using an easy, safe, and easily accessible method. Periodontitis is currently diagnosed using radiography and clinical measurements of probing pocket depth (PD), bleeding on probing (BOP), and clinical attachment level (CAL) (Salvi et al., 2008). However, these traditional clinical measurements are time-consuming and yield limited information because they are indicators of previous periodontal disease rather than present disease activity. Moreover, they are inadequate for predicting susceptible individuals who might be at risk of periodontitis in the future. The best predictor of gingival inflammation to date is BOP, but there are too many false positives associated with this method (Lang et al., 1990). There is an unmet need for near-patient testing to diagnose periodontitis.

Saliva is an optimal biological fluid to serve as the diagnostic tool for periodontitis. The collection of saliva is safe, non-invasive, and simple, and saliva can be collected repeatedly with minimum discomfort to the patient. A number of promising biomarkers have been already identified in saliva that correlate with the clinical parameters of periodontitis (Miller et al., 2010; AlMoharib et al., 2014; Taylor, 2014). Saliva contains locally produced proteins, genetic/genomic biomarkers such as DNA and mRNA, and various metabolites that originate from the host and the bacteria (Cuevas-Córdoba and Santiago-García, 2014). However, the diagnosis of periodontitis using saliva has a limitation in detecting disease activity at each individual tooth site; traditional clinical measurements are required in order to accomplish this. In this respect, the diagnosis of periodontitis using saliva must be realized as a point-of-care (POC) testing. POC testing is defined as medical testing conducted outside of a laboratory at or near the site of patient care, including the patient's bedside, the doctor's office, and the patient's home (Song et al., 2014). If periodontitis is diagnosed by a POC device using saliva, patients could easily diagnose their periodontitis at home and visit dental clinics at a suitable time. In dental clinics, current disease activity and responses to treatment can be easily monitored at a chair-side. A POC device for diagnosing periodontitis would also assist medical doctors in assessing the periodontal status of their patients because periodontitis is associated with many systemic diseases, such as atherosclerosis, coronary heart disease, diabetes mellitus, and rheumatoid arthritis (Scannapieco, 2005; Kobayashi and Yoshie, 2015). When medical doctors prescribe bisphosphonate or other medicines associated with medication-related osteonecrosis of the jaw (MRONJ), they can consider the periodontal status of their patients in advance to prevent the development of MRONJ, a common complication of medication combined with tooth extraction (Katsarelis et al., 2015). Recent developments in POC testing indicate that the diagnosis of periodontitis using saliva is now technically feasible.

## POC technologies for molecular diagnostics

Technologies for detecting biomarker signals in biofluids have advanced significantly. In particular, the combination of microfluidic and lab-on-a-chip technologies allows for real-time monitoring of biomarkers in a small volume of a bodily fluid at POC sites (Sackmann et al., 2014). Lab-on-a-chip approaches integrate processing steps such as sampling, sample preparation, detection, and data analysis into one small device (Su et al., 2015). Microfluidics-based devices can analyze diverse clinical samples, including blood, saliva, nasal aspirate, and urine (Su et al., 2015).

Diagnostic targets detected by POC technologies include nucleic acids, proteins, metabolites and other small molecules (Song et al., 2014; Su et al., 2015). For example, nucleic acid can be amplified by on-chip PCR (non-isothermal) or on-chip isothermal amplification techniques (Su et al., 2015). Many PCR-based POC devices for the detection of pathogens such as influenza, RSV, HIV, Methicillin-resistant *Staphylococcus aureus, Clostridium difficile*, and malaria are already commercially available (Su et al., 2015). POC DNA tests have also been developed to detect genetic mutations associated with various cancers (Yang et al., 2014). The microfluidic detection of protein biomarkers generally relies on antibody-based immunoassays. Aptamers, DNA, or RNA oligonucleotides designed to bind to various biomolecules with high specificity and sensitivity are an alternative to antibodies (Toh et al., 2015). The simple lateral flow assay is rapid and specific but not sensitive or quantitative. Diverse new technologies have been developed to improve sensitivities and to allow for quantitative measurements of multiplex protein biomarkers (Gaster et al., 2009; Warsinke, 2009; Rissin et al., 2010; de la Rica and Stevens, 2012). Glucose is the best-known metabolite targeted by POC testing, with a long history of use (Wilkins and Atanasov, 1996). Now a much wider range of analytes can be quantified using POC technology (Sia and Chin, 2011). For example, the i-STAT POC device, millions of which are sold annually, electrochemically measures blood gas (pH, *P*CO_2_, *P*O_2_, TCO_2_, HCO_3_, base excess, and sO_2_), electrolyte (sodium, potassium, chloride, TCO_2_, anion gap, ionized calcium, glucose, urea nitrogen, creatinine, and lactate), and hematology (hematocrit and hemoglobin) parameters (Lauks, 1998). A microfluidic device that measures nitric oxide has also been developed (Halpin and Spence, 2010).

Various approaches have been developed to detect the target molecules, but optical detection and electrochemical detection are the ones most commonly adopted. Optical detection methods implemented in POC devices include absorbance colorimetry, chemiluminescence, fluorescence, surface-enhanced Raman scattering spectroscopy, and surface plasmon resonance (Gubala et al., 2012; Su et al., 2015). Electrochemical detection methods include amperometric, potentiometric, and impedimetric measurements (Su et al., 2015).

## Salivary biomarkers of periodontitis

Ideal biomarkers of periodontitis must be able to (1) diagnose the presence of periodontal disease, (2) reflect the severity of the disease (3) monitor the response of the disease to treatment, and (4) predict the prognosis/progress of the disease. A number of biomarkers that satisfy at least one of the four requirements have been identified in saliva (Tables 1–**4**). Salivary biomarkers of periodontal disease can originate from both bacteria and the host. As periodontitis progresses, gingival inflammation, soft tissue destruction, and bone destruction occur sequentially and release associated proteins or metabolites into the saliva. Therefore, host-derived biomarkers are categorized according to whether they reflect inflammation, soft tissue destruction, or bone destruction. The biomarkers that satisfy three of the four requirements in at least three separate studies are classified as strong (S) biomarkers. When the number of studies that reported no difference or contradictory results is equal or greater than those with supporting results, the biomarkers are classified as questionable (Q). The remaining biomarkers are classified as potential (P).

**Table 1:** **Bacteria-derived salivary biomarkers**.

	**Salivary biomarkers**	**Supporting reports**		**No difference or contradictory reports**		**Strength**
		**References**	**Study size^†^**	**References**	**Study size**	
DNA	*Porphyromonas gingivalis*	von Troil-Lindén et al., 1995 (C); Sawamoto et al., 2005 (I)^*^; Ramseier et al., 2009 (C); Saygun et al., 2011 (C); Nomura et al., 2012 (L); Pereira et al., 2012 (I)	512			S
	*Prevotella intermedia*	von Troil-Lindén et al., 1995 (C); Ramseier et al., 2009 (C); Saygun et al., 2011 (C); Nomura et al., 2012 (L); Pereira et al., 2012 (I)	463			S
	*Tannerella forsythia*	Sawamoto et al., 2005 (C)^*^; Ramseier et al., 2009 (C); Saygun et al., 2011 (C); Pereira et al., 2012 (I)	387	Nomura et al., 2012 (L)	85	S
	*Treponema denticola*	Ramseier et al., 2009 (C); Pereira et al., 2012 (I)	188			P
	*Campylobacter rectus*	von Troil-Lindén et al., 1995 (C); Ramseier et al., 2009 (C); Saygun et al., 2011 (C)	289	Pereira et al., 2012 (I)	89	P
	*Pseudomonas aeruginosa* + *Acinetobacter* spp.	Souto et al., 2014 (C)	224			P
	*Peptostreptococcus micros*	von Troil-Lindén et al., 1995 (C)	40			P
	*Fusobacterium nucleatum*	Saygun et al., 2011 (C)	150	Ramseier et al., 2009 (C)	99	Q
	*Aggregatibacter actinomycetemcomitans*	von Troil-Lindén et al., 1995 (C); Saygun et al., 2011 (C)	190	Sawamoto et al., 2005 (C); Pereira et al., 2012 (I)	138	Q
Proteins	Dipeptidyl peptidase	Aemaimanan et al., 2009 (C)^*^	90			P

† Combined from multiple studies, C, cross-sectional study; L, longitudinal study; I, interventional study;

* *, correlation with clinical parameters; S, strong; P, potential; Q, questionable*.

### Bacteria-derived biomarkers

Bacteria-derived biomarkers include DNA and proteins. The levels of well-known pathogenic bacteria, such as *Aggregatibacter actinomycetemcomitans*, the three red complex species, and several species of the orange complex in saliva were determined by targeting a specific area of the 16S rRNA gene (Table 1). Among them, only *Porphyromonas gingivalis, Prevotella intermedia*, and *Tannerella forsythia* have been proved by multiple studies as strong biomarkers of periodontitis. Recent studies using high-throughput sequencing of the 16S rRNA gene have identified new species/phylotypes that are associated with periodontitis (Griffen et al., 2012; Göhler et al., 2014). Given the complexity of dental biofilm, the potential of the newly identified species/phylotypes to serve as salivary biomarkers of periodontitis needs to be investigated. The activity of dipeptidyl peptidase IV in saliva has been shown to be associated with periodontitis and the presence of *P. gingivalis* (Aemaimanan et al., 2009). Dipeptidyl peptidase IV is a serine protease that cleaves X-Pro dipeptide from the N-terminus of polypeptide chains, thus contributing to collagen degradation (Banbula et al., 2000). DPP4 in saliva may originate from both the host and bacteria, including *P. gingivalis* (Aemaimanan et al., 2009).

### Host-derived inflammatory biomarkers

Periodontitis begins with inflammation of the gingival tissue in response to dental biofilm. As inflammatory biomarkers in saliva, diverse enzymes (arginase, dipeptidyl peptidase IV, β-glucuronidase, and myeloperoxidase), anti-microbial proteins (lactoferrin and calprotectin), inflammatory cytokines (IL-1β, IL-6, IL-18, IFN-γ, and MIP-1α), and proteins that mediate inflammation (chemerin, CRP, TLR4, soluble CD14, and procalcitonin) have been studied (Table 2). Particularly, IL-1β, MIP-1α, and arginase are strong biomarkers that correlate with inflammatory parameters of periodontitis, such as the gingival index or BOP (Miller et al., 2006; Gheren et al., 2008; Al-Sabbagh et al., 2012; Rathnayake et al., 2013). In addition to protein biomarkers, nitric oxide, 8-hydroxydeoxyguanosine, platelet activating factor and fatty acid metabolites (neopterin, docosapentaenoate, linoleate, and arachidonate) have been identified as inflammation-associated biomarkers in saliva (Table 2).

**Table 2:** **Host-derived salivary biomarkers associated with inflammation**.

	**Salivary biomarkers**	**Supporting reports**		**No difference or contradictory reports**		**Strength**
		**References**	**Study size^†^**	**References**	**Study size**	
Proteins	IL-1β	Miller et al., 2006 (C)^*^; Ng et al., 2007 (C)^*^; Christodoulides et al., 2007 (C); Scannapieco et al., 2007 (L); Tobon-Arroyave et al., 2008 (C)^*^; Fine et al., 2009 (L)^*^; Gursoy et al., 2009 (C); Mirrielees et al., 2010 (C); Yoon et al., 2012 (C); Rathnayake et al., 2013 (C)^*^; Ebersole et al., 2013 (C); Fine et al., 2014 (L)	1396	Teles et al., 2009 (C); Ramseier et al., 2009 (C)	217	S
	MIP-1α	Fine et al., 2009 (L)^*^; Al-Sabbagh et al., 2012 (C)^*^; Fine et al., 2014 (L)	198			S
	Arginase	Ozmeriç et al., 2000 (C); Gheren et al., 2008 (I)^*^; Pereira et al., 2012 (I)	160			S
	soluble CD14	Isaza-Guzmán et al., 2008 (C)^*^; Prakasam and Srinivasan, 2014 (I)	110			P
	IFN-γ andIFN-γ/IL-22 ratio	Isaza-Guzmán et al., 2015 (C)^*^	149			P
	Lactoferrin	Fine et al., 2002 (C); Jentsch et al., 2004 (I); Glimvall et al., 2012 (C)^*^	79	Groenink et al., 1999 (C)	39	P
	Dipeptidyl peptidase	Aemaimanan et al., 2009 (C)^*^	90			P
	Chemerin	Özcan et al., 2015 (C)^*^	72			P
	Procalcitonin	Hendek et al., 2015 (C)^*^	72			P
	Calprotectin	Ramseier et al., 2009 (C)	99			P
	Myeloperoxidase	Meschiari et al., 2013 (C)	72			P
	IL-18	Banu et al., 2015 (C)	60			P
	TLR4	Banu et al., 2015 (C)	60			P
	β-glucuronidase	Lamster et al., 2003 (C)^*^; Yoon et al., 2012 (C)	497	Pietruska et al., 2006 (I)	16	P
	CRP	Pederson et al., 1995 (C); Christodoulides et al., 2007 (C); Shojaee et al., 2013 (C)^*^	186	Aurer et al., 2005 (C)	51	P
	IL-6	Costa et al., 2010 (C); Ebersole et al., 2013 (C); Prakasam and Srinivasan, 2014 (C)	210	Teles et al., 2009 (C); Gursoy et al., 2009 (C); Ramseier et al., 2009 (C); Rathnayake et al., 2013 (C); Khalaf et al., 2014 (C)	873	Q
	IL-8	Fine et al., 2014 (L)	70	Teles et al., 2009 (C); Rathnayake et al., 2013 (C)^*^; Khalaf et al., 2014 (C)	609	Q
	TNFα	Frodge et al., 2008 (C)	42	Teles et al., 2009 (C); Ramseier et al., 2009 (C); Gursoy et al., 2009 (C); Mirrielees et al., 2010 (C); Ebersole et al., 2013 (C)	567	Q
Metabolites	Nitric oxide	Reher et al., 2007 (C)^*^; Khorsavi Samani et al., 2012 (C); Parwani et al., 2012 (I)^*^; Han et al., 2013 (C); Sundar et al., 2013 (C)	466			S
	8-hydroxydeoxyguanosine	Sugano et al., 2003 (I); Sawamoto et al., 2005 (I); Takane et al., 2005 (C); Canakçi et al., 2009a (C); Canakçi et al., 2009b (C); Sezer et al., 2012 (C)^*^	297			S
	Platelet activating factor	McManus and Pinckard, 2000 (C)	165			P
	Neopterin	Ozmeriç et al., 2002 (C)	30			P
	ω-3 (docosapentaenoate) and ω-6 (linoleate and arachidonate) fatty acids	Barnes et al., 2014 (C)	80			P

† Combined from multiple studies, C, cross-sectional study; L, longitudinal study; I, interventional study;

* *, correlation with clinical parameters; S, strong; P, potential; Q, questionable*.

### Host-derived biomarkers associated with soft tissue destruction

As periodontitis progresses, soft tissues are destroyed, releasing several enzymes and proteins that are involved in tissue destruction into the saliva. Among them, MMP-8, MMP-9, HGF, lactate dehydrogenase, aspartate aminotransferase, and TIMP-2 are strong or potential biomarkers of periodontitis (Table 3). In addition, a recent metabolomic profiling of saliva revealed increased amounts of metabolites originated from macromolecular degradation, including dipeptides (proteins), oligo/mono-saccharides (polysaccharides), lysolipids, fatty acids, and monoacylglycerol (glycerophospholipid and triacylglycerol), and uridine (DNA/RNA) in periodontitis (Table 3).

**Table 3:** **Host-derived salivary biomarkers associated with soft tissue destruction**.

	**Salivary biomarkers**	**Supporting reports**		**No difference or contradictory reports**		**Strength**
		**References**	**Study size^†^**	**References**	**Study size**	
Protein	MMP-8	Gorska and Nedzi-Gora, 2006(I); Miller et al., 2006 (C)^*^; Christodoulides et al., 2007 (C); Ramseier et al., 2009 (C); Mirrielees et al., 2010 (C); Costa et al., 2010 (C); Gursoy et al., 2010 (C); Gursoy et al., 2013 (C); Ebersole et al., 2013 (C); Meschiari et al., 2013 (I); Rathnayake et al., 2013 (C)^*^	1371			S
	HGF	Wilczynska-Borawska et al., 2006 (C)^*^; Scannapieco et al., 2007 (L); Rudrakshi et al., 2011 (C)^*^; Lönn et al., 2014 (C)	222			S
	Aspartate aminotransferase	Nomura et al., 2006 (C); Totan et al., 2006 (C); Nomura et al., 2012 (L); Banu et al., 2015 (C)	382			P
	Lactate dehydrogenase	de la Peña et al., 2005 (I); Nomura et al., 2006 (C); Kugahara et al., 2008 (C); Nomura et al., 2012 (L)	568	Gursoy et al., 2009 (I)	165	P
	MMP-9	Ramseier et al., 2009 (C); Isaza-Guzmán et al., 2011 (C)^*^; Gursoy et al., 2013 (C)	452	Gorska and Nedzi-Gora, 2006 (I)	40	P
	TIMP-2	Meschiari et al., 2013 (I)	72			P
	Alanine aminotransferase	Nomura et al., 2012 (L); Banu et al., 2015 (C)	145	Nomura et al., 2006 (C); Totan et al., 2006 (C)	237	Q
	TIMP-1	Gursoy et al., 2010 (C); Isaza-Guzmán et al., 2011 (C)^*^	288	Hayakawa et al., 1994 (C); Gorska and Nedzi-Gora, 2006 (I); Meschiari et al., 2013 (I); Rathnayake et al., 2013 (C)^*^	573	Q
Metabolites	Purine degradation metabolites (e.g., guanosine and inosine)	Barnes et al., 2014 (C)	80			P
	Dipeptide, amino acid, carbohydrate, lipids, and nucleotide metabolites	Barnes et al., 2011 (C)	68			P

† Combined from multiple studies, C, cross-sectional study; L, longitudinal study; I, interventional study;

* *, correlation with clinical parameters; S, strong; P, potential; Q, questionable*.

### Host-derived biomarkers associated with bone destruction

Salivary biomarkers of bone remodeling can be used as indicators of bone destruction in periodontitis. These include alkaline phosphatase, osteonectin, RANKL, and calcium (Table 4). A positive correlation between the levels of salivary calcium and CAL has been reported (Sutej et al., 2012).

**Table 4:** **Host-derived salivary biomarkers associated with hard tissue destruction**.

	**Salivary biomarkers**	**Supporting reports**		**No difference or contradictory reports**		**Strength**
		**References**	**Study size^†^**	**References**	**Study size**	
Protein	Alkaline phosphatase	Totan et al., 2006 (C); Kugahara et al., 2008 (C); Dabra and Singh, 2012 (C)	331	Nomura et al., 2012 (L)	85	P
	Osteonectin	Scannapieco et al., 2007 (L); Ng et al., 2007 (C)^*^	80			P
	RANKL	Buduneli et al., 2008 (C); Tobón-Arroyave et al., 2012 (C)^*^	195	Frodge et al., 2008 (C)	42	P
	Osteoprotegerin	Ramseier et al., 2009 (C); Tobón-Arroyave et al., 2012 (C)^*^; Hassan et al., 2015 (I)	269	Miller et al., 2006 (C)^*^; Buduneli et al., 2008 (C); Costa et al., 2010 (C); Al-Sabbagh et al., 2012 (C)^*^	282	Q
Metabolites	Calcium	Acharya et al., 2011 (C); Sutej et al., 2012 (C)^*^	67			P
	Pyridinoline cross-linked carboxyterminal telopeptide of type I collagen	Gursoy et al., 2010 (C)	165	Frodge et al., 2008 (C); Ramseier et al., 2009 (C); Al-Sabbagh et al., 2012 (C); Gursoy et al., 2013 (C)	386	Q

† Combined from multiple studies, C, cross-sectional study; L, longitudinal study; I, interventional study;

* *, correlation with clinical parameters; S, strong; P, potential; Q, questionable*.

### Further considerations

Among the various salivary biomarkers listed, *P. gingivalis* has been shown to satisfy all four requirements of ideal biomarkers for periodontitis in at least one study for each requirement. However, single biomarker detection may not be effective enough for accurate diagnoses without false-positive or false-negative results. Periodontitis is a disease that involves complex interactions between bacteria and the host immune system. The combination of the host-derived biomarkers, which reflect inflammation, soft tissue destruction, and bone destruction together with bacteria-derived biomarkers, may be useful to diagnose not only the presence of periodontitis but also the degree of progression and the response to therapy. A number of host-derived biomarkers have already shown strong associations with periodontitis.

The fact that the concentration of biomarkers can be affected by the saliva flow rate, circadian rhythm, age, the physiological status of the patients, and other factors raises concern over the accuracy and reproducibility of diagnoses using salivary biomarkers (Nový, 2014). Although within-subject correlations between unstimulated and stimulated samples and over time have been reported for some salivary proteins (Rudney et al., 1985), such studies have not been done for all salivary proteomes or metabolomes. Nevertheless, many biomarkers, in numerous studies, have shown consistent associations with periodontitis. For example, significantly higher levels of salivary MMP-8 in periodontitis than in healthy controls were observed in six studies that used the non-stimulatory whole saliva samples (Miller et al., 2006; Christodoulides et al., 2007; Ramseier et al., 2009; Costa et al., 2010; Mirrielees et al., 2010; Ebersole et al., 2013) and in five studies that used stimulated whole saliva samples (Gorska and Nedzi-Gora, 2006; Gursoy et al., 2010, 2013; Meschiari et al., 2013; Rathnayake et al., 2013). These findings suggest that within-subject variations in the concentration of salivary biomarkers can be overcome for diagnostic purposes if a biomarker with substantial inter-group differences is chosen.

## POC devices in periodontology

A few POC devices have been developed for the salivary diagnosis of periodontitis. A device called the Integrated Microfluidic Platform for Oral Diagnostics (IMPOD) was able to detect salivary proteins with a low sample volume requirements (10 μL) and considerable sensitivity (nM-pM) by integrating sample pretreatment (filtering, enrichment, mixing) with electrophoretic immunoassays and a laser-induced fluorescence detection system. Using this device, rapid (< 10 min) measurements of MMP-8, TNF-α, IL-6, and CRP in saliva were performed (Herr et al., 2007a,b). However, validation in the clinical setting has not yet been reported.

A group at the University of Texas at Austin developed a lab-on-a-chip (LOC) system that integrates microfluidics and a fluorescence-based optical system in which sandwich immunoassays are performed on chemically sensitized beads. They reported the application of the LOC system for the multiplex measurement of three salivary biomarkers, C-reactive protein, MMP-8, and IL-1β, which are related to the clinical expression of periodontitis. The LOC approach yielded a limit of detection five orders of magnitude lower than that of a standard ELISA, and the results obtained by the LOC approach were consistent with ELISA results (Christodoulides et al., 2007). Whether the POC device using this LOC approach can accurately measure levels of the salivary biomarker MMP-8 and thus indicate if a patient has periodontal health, gingivitis or periodontal disease is currently being studied in a clinical trial (NCT02403297 at ClinicalTrials.gov).

## Suggestions for organizing the international consortium for salivary biomarkers of periodontitis

For the success of POC diagnostics of periodontitis using saliva, it is important to validate the candidate biomarkers with large populations which suitably account for diversity such as those related to race, region, gender, and age. In periodontal research, full-mouth or selected teeth have been examined to diagnose periodontitis, and different criteria have been also used to classify the severity of periodontitis. Such variations in the classifications of patients make it difficult to integrate the results of different studies. Furthermore, variations in saliva sampling methods (e.g., resting vs. stimulatory, whole vs. individual gland sampling), target biomarkers, and detection methods of salivary biomarkers prevent direct comparisons of the data obtained from different studies or centers. We propose the organization of an International Consortium for Salivary Biomarkers of Periodontitis (ICSBP). The ICSBP can put forth collaborative efforts to create standardized protocols for clinical research, including a uniform method for clinical diagnoses of periodontitis. In addition, the ICSBP can accelerate the validation of biomarkers and the implementation of salivary diagnostics by sharing clinical samples and experience.

## Funding

This work was supported by the National Research Foundation of Korea (Daejun, Korea) Grant 2014050477 funded by the Korean Government through the Oromaxillofacial Dysfunction Research Center for the Elderly at Seoul National University and by Basic Science Research Program through the National Research Foundation grants 2013R1A1A3005669 and 2014R1A1A1005507.

### Conflict of interest statement

The authors declare that the research was conducted in the absence of any commercial or financial relationships that could be construed as a potential conflict of interest.
